# Effects of Rumen-Protected Taurine Supplementation on Ruminal Fermentation, Hematological Profiles, Liver Function, and Immune Responses in Yaks

**DOI:** 10.3390/ani15131929

**Published:** 2025-06-30

**Authors:** Shoupei Zhao, Lianghao Lu, Yuanyuan Chen, Huaming Yang, Bao Zhang, Mingyu Cao, Wenju Chao, Wanchao Xue, Xiaorong Fan, Jianxin Xiao, Rui Hu, Quanhui Peng, Lizhi Wang, Zhisheng Wang, Bai Xue

**Affiliations:** 1 Animal Nutrition Institute, Sichuan Agricultural University, Chengdu 611130, China; 2023114011@stu.sicau.edu.cn (S.Z.); 13551012639@163.com (L.L.); cyy2022214041@stu.sicau.edu.cn (Y.C.); 13980277829@163.com (H.Y.); 2022314074@stu.sicau.edu.cn (B.Z.); 2021114018@stu.sicau.edu.cn (M.C.); xiaojianxin@sicau.edu.cn (J.X.); ruitianhu@yeah.net (R.H.); pengquanhui@126.com (Q.P.); wanglizhi08@aliyun.com (L.W.); wangzs67@163.com (Z.W.); 2 Huangyuan County Animal Husbandry and Veterinary Station, Xining 812100, China; m18884771172@163.com (W.C.); z15530273022@126.com (W.X.); 3 Ping’an District Agriculture and Technology Bureau, Haidong 810799, China; zz1999996@163.com

**Keywords:** yaks, rumen-protected taurine, humoral immunity, liver function, rumen fermentation, hematological parameters

## Abstract

The present study evaluated the effects of dietary rumen-protected taurine (RPT) supplementation on ruminal fermentation, hematological parameters, liver function, stress-related hormones, and immune responses in yaks. Eighteen yaks were randomly allocated to three groups: a control group receiving no RPT (CON), a low-dose group receiving 20 g/day (RPT20), and a high-dose group receiving 40 g/day (RPT40). Supplementation with RPT did not significantly affect ruminal pH, microbial protein concentration, ammonia nitrogen, total volatile fatty acids, or the individual volatile fatty acid profiles (*p* > 0.05). A decreasing trend in red blood cell count was observed (*p* = 0.050), while no significant changes were detected in white blood cell or platelet indices (*p* > 0.05). Liver function markers, including albumin, alanine transaminase, aspartate transaminase, and total protein, remained unchanged, although a trend toward altered alkaline phosphatase activity was noted (*p* = 0.074). No significant effects were observed on acute-phase proteins (serum amyloid A, C-reactive protein) or stress-related hormones (epinephrine, adrenocorticotropic hormone, cortisol) (*p* > 0.05). Importantly, serum immunoglobulin A and immunoglobulin G levels were significantly increased in response to RPT supplementation (*p* = 0.029 and *p* = 0.043, respectively), suggesting enhanced humoral immunity. These findings indicate that RPT may improve immune function in yaks without negatively affecting rumen fermentation or liver health.

## Simple Summary

Yaks are essential to the livelihoods of herders on the Qinghai–Tibet plateau, but the transition to off-site intensive fattening systems has raised concerns about their nutritional and immune health. Due to environmental factors, such as altitude, temperature fluctuations, and dietary changes, yaks are susceptible to stress and compromised immune function. Taurine, a nutrient known for its immune-boosting and stress-resilience properties, is rapidly degraded in the rumen, limiting its availability. This study investigated the effects of rumen-protected taurine (RPT) supplementation on yaks. The results showed that RPT supplementation did not significantly alter ruminal fermentation or liver function. However, it significantly increased the serum levels of immune proteins IgA and IgG. Additionally, RPT supplementation was associated with changes in red blood cell counts, which may reflect an adaptive response to the high-altitude environment. These findings suggest that RPT supplementation can enhance immune function in stall-fed yaks without negatively impacting liver health.

## 1. Introduction

Yaks (*Bos grunniens*) are a cornerstone of the pastoral economy on the Qinghai–Tibet plateau, supplying milk, meat, wool, fuel, and transport services to local communities [1]. As the only bovine species capable of long-term survival at altitudes exceeding 3000 m, yaks are uniquely adapted to high-altitude environments and play a critical role in sustaining rural livelihoods [2]. However, traditional grazing systems are increasingly challenged by prolonged cold seasons, forage shortages, and the degradation of natural grasslands, all of which contribute to nutritional imbalances, reduced productivity, and weakened immune resilience in yaks [3,4].

To address these issues, a transition from extensive grazing to off-site intensive fattening systems has been widely adopted. Stall feeding enables more precise dietary formulation and nutrient delivery. However, yaks may experience stress responses and reduced immune function due to significant variations in altitude, temperature, and diet [5,6]. One such nutrient of growing interest is taurine, a biologically active amino acid known for its roles in bile salt conjugation, membrane stabilization, osmoregulation, calcium signaling, antioxidation, and immune modulation [7]. Taurine is present in metabolically active tissues, such as in leukocytes, skeletal muscle, the retinas, the brain, and the heart. Although most mammals synthesize taurine endogenously, the levels produced are often inadequate under conditions of physiological stress or rapid growth, making dietary supplementation essential [8]. Since the ban on animal-derived feedstuffs in ruminant nutrition following the 1980s outbreak of bovine spongiform encephalopathy (BSE) [9], plant-based diets have become the norm. However, as shown by Spitze et al. [10], plant-derived feed ingredients are virtually devoid of taurine, creating a critical gap in nutrient supply for ruminants. Despite taurine’s known benefits in monogastric animals, its application in ruminants, especially yaks, remains largely unexplored due to its extremely high ruminal degradation rate—over 99% within two hours [11].

To mitigate this challenge, rumen-protected technologies have been developed to shield sensitive nutrients from ruminal breakdown and ensure their post-ruminal release and absorption [12]. These technologies, including encapsulation and chemical modification, have been successfully applied to improve amino acid bioavailability and performance traits in cattle and yaks, such as enhancing growth, milk yield, and meat quality via protected methionine and lysine [13,14].

While recent evidence suggests that rumen-protected taurine (RPT) may enhance protein turnover and support growth in other species [15], its effects on yaks—particularly in relation to rumen fermentation, hematological parameters, liver function, oxidative stress, and immune status—have not been systematically investigated. Given the physiological demands placed on yaks in high-altitude, cold-stress environments, and their nutritional vulnerability under stall-feeding conditions, understanding the role of RPT in yak health and metabolism is both timely and necessary.

Therefore, the present study was conducted to evaluate the effects of rumen-protected taurine supplementation on rumen fermentation parameters, hematological profiles, liver health, serum stress biomarkers, and immune indices in yaks. This research aims to fill a critical knowledge gap and provide scientific evidence supporting the nutritional management of yaks under modern feeding systems.

## 2. Materials and Methods

### 2.1. Animals and Experimental Design

The experiment was conducted from April to May 2023 at the Teaching and Research Base of Sichuan Agricultural University in Ya’an, Sichuan Province (E 102°59′2″, N 29°58′59″), located at an elevation of 546.3 m. Eighteen male yaks (184.97 ± 4.02 kg) were randomly assigned to three groups, with six yaks per group. Each yak was housed in an individual pen (1.5 m × 2 m) and tethered. All groups were fed the same total mixed ration (TMR) as a basic diet, supplemented with varying levels of RPT: 0 g/d (CON), 20 g/d (RPT20), and 40 g/d (RPT40). The RPT used in the experiment was produced by Qingdao Kailede Biotechnology Co., Ltd. It contained 50% taurine, and its coating consisted of 30% stearic acid and 20% excipients, including microcrystalline cellulose, starch, and methylcellulose. The in vitro rumen bypass rate of RPT was determined to be 95.7% using the first step of the Tilley and Terry two-stage digestion method [6]. The basic diet was formulated according to the Nutrient Requirements of Beef Cattle (NASEM, 2016) [16]. The TMR composition and nutritional components are shown in Table 1. TMR samples were collected every 5 days using a multi-point sampling method (six points per sampling), stored at −20 °C, and pooled for nutrient analysis. The nutrient composition of the TMR was determined following our previous research [17]. Briefly, DM was determined according to AOAC (2005; methods 930.15) [18]. Nitrogen content was analyzed using the Kjeldahl method (AOAC, 2005; method 981.10) [18] and converted to crude protein (CP) as N × 6.25. Calcium and phosphorus were analyzed by flame atomic absorption spectrometry and fluorescence spectrophotometry, respectively (AOAC, 1990; methods 985.35 and 986.24) [19]. Neutral detergent fiber (NDF) and acid detergent fiber (ADF) were determined according to Van Soest et al. (1991) [20]. The values of net energy for maintenance (NEm) and net energy for gain (NEg) were calculated based on the equations of NASEM (2016) [16].

The trial lasted 44 days, consisting of a 14-day pre-feeding period and a 30-day formal feeding period. Prior to the trial, the barn was disinfected, and each yak was numbered and dewormed. Yaks were fed twice daily at 08:00 and 18:00. At each feeding event, 50% of the daily dosage of RPT was homogeneously incorporated into a small fraction of the TMR and administered as the initial feed offering. Upon complete consumption of this additive-enriched fraction, the remaining basal TMR was subsequently provided. Approximately 10% of the total daily ration was left as refusal. Water was provided ad libitum throughout the trial.

### 2.2. Rumen Fluid Collection and Analysis

Rumen fluid was collected on d 30, prior to the morning feeding. Each yak was immobilized in a single stall using a neck clamp, and the distance from the head to the rumen was measured to determine the appropriate depth for the stomach tube. A rumen fluid collection tube (2.6 m in length, approximately 19 mm in outer diameter, with a sampling head outer diameter of 30.04 mm; Kolibo Co., Ltd., Hangzhou, China) was selected. A total of 50 mL of rumen fluid was collected, with the first 10 mL discarded prior to each collection.

The remaining fluid was filtered through four layers of gauze, and the pH was measured immediately using a pH meter (S-10, HORIBA Ltd., Osaka, Japan). The filtered rumen fluid was transferred into 10 mL centrifuge tubes and stored at −20 °C for subsequent analyses. Two subsamples were separately preserved with 2 mL of 25% (wt/vol) metaphosphoric acid and 2 mL of 1% (wt/vol) sulfuric acid for the analysis of total volatile fatty acids (TVFAs) and ammonia nitrogen (NH_3_-N), respectively. The TVFA, including acetic acid, propionic acid, butyric acid, isobutyric acid, valeric acid, and isovaleric acid, were quantified using a gas chromatograph (Agilent 8890 GC, Santa Clara, CA, USA) [21]. NH_3_-N concentration was determined using an F-7000 fluorescence spectrophotometer (Hitachi, Ltd., Tokyo, Japan) by the phenol-hypochlorite colorimetric method [22]. Microbial protein (MCP) was quantified using a commercial BCA protein assay kit (Solabio, Beijing, China) [23].

### 2.3. Serum Sample Collection and Analysis

Before the morning feeding on d 30 of the experiment, blood samples were collected from all the yaks in each group via the jugular vein into evacuated tubes containing sodium heparin (for whole blood analysis) and into those without an anticoagulant (for serum analysis). Whole blood in heparinized tubes was immediately analyzed using a fully automated blood cell analyzer (BC-5000, Mindray, Shenzhen, China) for white blood cell, red blood cell, and platelet parameters. Blood samples in non-anticoagulant tubes were allowed to stand for 30 min, then centrifuged at 3000× *g* for 20 min at 4 °C. The serum was stored at −80 °C for biochemical measurements and metabolomics.

Serum levels of albumin (ALB), alkaline phosphatase (ALP), alanine transaminase (ALT), aspartate transaminase (AST), total protein (TP), and blood urea nitrogen (BUN) were measured using an automated biochemical analyzer (Hitachi 3100, Hitachi High-Technologies Corporation, Tokyo, Japan).

Serum levels of interleukins (IL-1, IL-6), serum amyloid A (SAA), tumor necrosis factor (TNF-α, IFN-γ), C-reactive protein (CRP), immunoglobulins (IgA, IgG, IgM), epinephrine (EPI), adrenocorticotropic hormone (ACTH), and cortisol (COR) were measured using enzyme-linked immunosorbent assay (ELISA) kits (Jiangsu Meimian Industrial Co., Ltd., Jiangsu, China). The optical density (OD) was measured with an enzyme-labeled instrument (SpectraMax190, Molecular Devices, Sunnyvale, CA, USA).

### 2.4. Statistical Analysis

The experimental data were analyzed using one-way analyses of variance (ANOVA) followed by Duncan’s multiple comparisons in SPSS 17.0 statistics software (SPSS Inc. Chicago, IL, USA):

*Y_ij_* = *μ* + *α_i_* + *e_ij_*,

where *Y_ij_* is the dependent variable, *μ* is the overall mean, *α_i_* is the effect of the *i*-th group, and *e_ij_* is the random error. Results were presented as means and standard errors. Significance was considered at *p* < 0.05, with very significant differences at *p* < 0.01, and a trend observed for 0.05 ≤ *p* ≤ 0.10.

## 3. Results

### 3.1. Ruminal Fermentation Parameters

The effects of RPT supplementation on ruminal fermentation parameters in yaks are presented in Table 2. Supplementation with varying doses of RPT had no significant effect on ruminal pH or the concentrations of MCP, NH_3_-N, TVFA, acetic acid, propionic acid, butyric acid, isobutyric acid, valeric acid, and isovaleric acid, or the A/P ratio (*p* > 0.05).

### 3.2. Hematological Profiles

The addition of RPT to the diet had no significant effect on white blood cell or platelet levels in yak (*p* > 0.05) (Table 3). However, a trend toward a reduction in red blood cell count was observed (*p* = 0.050). No significant effects were found on the hematocrit, mean corpuscular volume, mean corpuscular hemoglobin, mean corpuscular hemoglobin concentration, or hemoglobin levels (*p* > 0.05).

### 3.3. Serum Biochemical Parameters Related to Liver Health

The effects of supplementing different doses of RPT on serum biochemical indicators related to liver function in yaks are summarized in Table 4. A trend towards an effect on ALP was also noted with RPT supplementation (*p* = 0.074). No significant effects were found on the serum levels of ALB, ALT, AST, and TP (*p* > 0.05).

### 3.4. Neuroendocrine and Acute-Phase Markers

There were no significant effects were found on the acute-phase proteins SAA and CRP (*p* > 0.05), nor on hormones such as EPI, ACTH, and COR (*p* > 0.05) (Table 5).

### 3.5. Serum Immunological and Inflammatory Indicators

The effects of the RPT supplementation on serum immune function indicators are presented in Table 6. Supplementation with RPT significantly influenced the serum concentrations of IgA (*p* = 0.029) and IgG (*p* = 0.043). The IgA concentration in the RPT20 group was significantly higher than that in the CON and RPT40 groups (*p* < 0.05), and the IgG concentration was significantly higher than that in the CON (*p* < 0.05). However, no significant differences were observed among the groups for the serum concentrations of IL-1, IL-6, TNF, and IFN-γ (*p* > 0.05).

## 4. Discussion

Taurine, a sulfur-containing semi-essential amino acid, has been widely utilized as an additive in livestock and poultry feed due to its roles in regulating antioxidant activity, lipid metabolism, and protein phosphorylation [24]. However, its effects on ruminants have not been thoroughly studied, primarily due to its rapid degradation in the rumen [11].

The rumen, a unique organ in ruminants, plays a central role in their survival by providing unmatched ecological resource use efficiency. Maintaining its homeostasis is essential for sustaining the health and production performance of ruminants. A comparison of the effects of protected versus unprotected amino acids on rumen fermentation parameters has shown that unprotected amino acids, such as leucine, are released excessively in the rumen, altering microbial composition and promoting the conversion of branched-chain amino acids to branched-chain VFAs, thus increasing branched-chain VFA and MCP production [25]. In the current study, the rumen bypass rate of RPT was 95.7%. Taurine was encapsulated in coating materials, resulting in low degradation rates in the rumen and no significant effects on rumen fermentation parameters. This suggests that RPT remains intact until it reaches the intestine, where it can exert its beneficial effects.

Hematological profiles are an important indicator of animal health, with changes in white blood cell count reflecting the body’s overall health and its ability to resist disease. In this study, no significant changes were observed in white blood cell levels, nor were any effects noted on lymphocytes, monocytes, or neutrophils. However, a decreasing trend in red blood cell count was observed with the addition of RPT. Previous studies on yaks have shown that red blood cell counts typically range from 9 to 11 × 10^12^/L in yaks raised at high altitudes [26]. As altitude decreases and oxygen levels increase, red blood cell counts generally decrease [27]. The experimental site, with sufficient oxygen and located at an altitude of 600 m, may explain the reduced demand for red blood cell count. Liu et al. [28] proposed that maintaining lower hemoglobin levels in yaks exposed to low-altitude heat stress helps reduce blood viscosity and increase blood flow velocity, thereby improving oxygen supply and heat dissipation. In this study, a decreasing trend in hemoglobin levels was observed alongside a reduction in red blood cell count. These findings suggest that RPT supplementation may enhance blood circulation, enabling the body to maintain an adequate oxygen supply and support nutrient metabolism despite the lower levels of red blood cells and hemoglobin.

Blood biochemistry can be used to assess the physiological and metabolic status of animals, reflecting organ function, nutritional status, and metabolic abnormalities. The ALT and AST are key indicators of liver function, reflecting the status of protein metabolism and hepatic protein synthesis in animals. In healthy ruminants, elevated levels of ALT and AST are typically associated with increased hepatic protein turnover [29]. However, when liver tissue is damaged, ALT and AST can be released from the cytoplasm of hepatocytes into the bloodstream, leading to elevated serum concentrations of these enzymes [30,31]. In this study, supplementation with RPT did not result in significant changes in liver function markers, such as TP, ALB, ALT, and AST. This suggests that RPT supplementation did not cause any damage to the liver.

The effects of dietary RPT supplementation on serum cytokines (IL-1, IL-6, TNF-α, IFN-γ), neuroendocrine and acute-phase markers (SAA, CRP, EPI, ACTH, and COR), and immunoglobulins (IgA, IgG, IgM) in yaks were also assessed to investigate RPT’s impact on immune performance. Cytokines, produced by various cells, are multifunctional proteins that regulate immune responses. Inflammation, triggered by external stimuli, leads to the production of pro-inflammatory cytokines, initiating a stress response in the body [32]. Taurine has been shown to reduce the concentration of IL-6 and TNF-α induced by lipopolysaccharide (LPS) in mice [33], and supplementation in broiler chickens can lower the expression of LPS-induced IL-1β, IL-6, and TNF-α [34,35], suggesting an inhibitory effect on pro-inflammatory factors. In this experiment, RPT supplementation did not significantly affect IL-1 levels but reduced IL-6 by 7.8% and 9.5% in the RPT20 and RPT40 groups, respectively, and decreased TNF-α by 4.3% and 31.8%. Acute-phase proteins, such as SAA and CRP, are essential components of innate immune defense, and play significant roles in inflammation regulation [35]. Both SAA and CRP were unaffected by taurine supplementation in this study. Epinephrine, ACTH, and cortisol constitute key elements of the neuroendocrine stress response, orchestrating both acute and chronic physiological adaptations through the regulation of cardiovascular function, energy metabolism, and immune activity [36]. However, supplementation with rumen-protected taurine had no significant effect on these indicators, possibly due to the favorable rearing conditions that did not induce substantial stress in the yaks. Immunoglobulins, produced by B lymphocytes, reflect the body’s health and immune status, comprising 15–20% of total serum proteins [37]. Supplementing taurine in fish diets has significantly increased immunoglobulin levels [38], and in broilers, replacing methionine with taurine elevated immunoglobulin levels, particularly IgG and IgA [39]. These findings align with our results, which showed that RPT20 significantly increased serum IgG and IgA, thereby improving the immune response in yaks.

## 5. Conclusions

This study explored the effects of rumen-protected taurine supplementation on various health and metabolic parameters in yaks. The results of this study indicate that—while rumen-protected taurine supplementation does not significantly impact rumen fermentation parameters, liver function, or stress responses in yaks—it can enhance immune function, particularly by increasing the serum concentrations of IgA and IgG. The observed reduction in red blood cells could be a natural adaptive response to the low-oxygen environment at high altitudes. These findings suggest that RPT may be a useful dietary supplement for improving immune health in yaks, especially under the off-site intensive fattening systems. Further research is needed to fully understand the mechanisms through which taurine supplementation influences yak health and to explore potential long-term benefits.

## Figures and Tables

**Table 1:** Ingredients and nutrient levels of diets (DM basis %).

Item	Contents
Ingredients	
Corn	20.15
Wheat bran	5.50
Soybean meal	15.30
Rapeseed meal	1.50
Wheat straw	30.00
Corn stalk silage	25.00
NaHCO_3_	1.00
CaCO_3_	0.60
Sodium chloride	0.50
Premix ^1^	0.45
Nutrient levels ^2^	
NEm, MJ/kg	5.36
NEg, MJ/kg	3.03
DM	59.45
CP	11.30
NDF	47.09
ADF	21.62
Ca	0.48
P	0.25

NEm = net energy for maintenance; NEg = net energy for gain; NDF = neutral detergent fiber; ADF = acid detergent fiber; CP = crude protein; Ca = calcium; P = phosphorus. ^1^ The premix provided the following nutrients per kilogram of the diets: vitamin A 4400 IU, vitamin D 600 IU, vitamin D 120 IU, Cu 9 mg, Fe 110 mg, Mn 43 mg, Zn 60 mg, Se 0.3 mg, I 0.6 mg, and Co 0.2 mg. ^2^ NEm and NEg were calculated from data provided by the Nutrient Requirements of Beef Cattle (NASEM, 2016) [16], and other values were analyzed.

**Table 2:** Effects of dietary supplementation with RPT on ruminal fermentation parameters in yaks.

Item	Treatment ^1^			SEM	*p*-Value
	CON	RPT20	RPT40		
pH	7.65	7.64	7.58	0.0223	0.408
MCP, mg/mL	1.86	1.91	2.11	0.0938	0.543
NH_3_-N, ug/mL	24.4	25.2	22.7	1.07	0.651
TVFA, mmol/L	63.4	57.3	60.8	2.85	0.711
Acetic acid, mmol/L	44.1	40.4	42.6	1.96	0.765
Propionic acid, mmol/L	13.9	12.6	13.5	0.713	0.751
Butyric acid, mmol/L	3.47	2.77	2.83	0.379	0.729
Isobutyric acid, mmol/L	0.806	0.640	0.826	0.0400	0.110
Valeric acid, mmol/L	0.248	0.165	0.206	0.0201	0.255
Isovaleric acid, mmol/L	0.868	0.806	0.847	0.0432	0.853
A/P	3.16	3.08	3.20	0.0472	0.765

RPT = rumen-protected taurine; MCP = microbial protein; NH_3_-N = ammonia nitrogen; TVFA = volatile fatty acids; A/P = acetic acid/propionic acid. *n* = 6. ^1^ CON means a basic diet without RPT; RPT20 means a basal diet with daily supplementation of 20 g of RPT; RPT40 means a basal diet with daily supplementation of 40 g of RPT.

**Table 3:** Effects of dietary supplementation with RPT on routine blood parameters in yaks.

Item	Treatment ^1^			SEM	*p*-Value
	CON	RPT20	RPT40		
White blood cell					
White blood cell, ×10^9^/L	9.96	9.16	8.89	0.460	0.640
Neutrophil count, ×10^9^/L	5.07	4.71	4.19	0.345	0.252
Lymphocyte count, ×10^9^/L	3.69	3.42	3.43	0.213	0.285
Monocyte count, ×10^9^/L	0.673	0.496	0.583	0.0403	0.139
Eosinophil count, ×10^9^/L	0.533	0.543	0.677	0.0417	0.284
Red blood cell					
Red blood cell count, ×10^12^/L	6.31	5.57	4.51	0.311	0.0502
Hematocrit, %	34.1	30.5	26.2	1.54	0.107
Mean corpuscular volume, fL	54.2	54.6	58.1	0.913	0.165
Mean corpuscular hemoglobin, pg	19.9	19.9	21.2	0.347	0.234
Mean corpuscular hemoglobin concentration, g/L	367	366	364	2.30	0.884
Hemoglobin, g/L	125	113	95.2	5.89	0.104
Platelet					
Platelet count, ×10^9^/L	312	297	295	26.4	0.963
Platelet distribution width	15.7	15.8	15.9	0.105	0.847

RPT = rumen-protected taurine. *n* = 6. ^1^ CON means a basic diet without RPT; RPT20 means a basal diet with daily supplementation of 20 g of RPT; RPT40 means a basal diet with daily supplementation of 40 g of RPT.

**Table 4:** Effects of dietary supplementation with RPT on antioxidant capacity in yaks.

Item	Treatment ^1^			SEM	*p*-Value
	CON	RPT20	RPT40		
ALB, g/L	28.9	27.9	26.5	0.535	0.203
ALP, U/L	103.5	110.5	82.7	5.29	0.0742
ALT, U/L	84.2	94.3	77.0	4.67	0.331
AST, U/L	69.5	66.2	75.2	4.58	0.744
TP, g/L	75.0	74.6	73.9	1.04	0.918

RPT = rumen-protected taurine; ALB = albumin; ALP = alkaline phosphatase; ALT = alanine transaminase; AST = aspartate transaminase; TP = total protein. *n* = 6. ^1^ CON means a basic diet without RPT; RPT20 means a basal diet with daily supplementation of 20 g of RPT; RPT40 means a basal diet with daily supplementation of 40 g of RPT.

**Table 5:** Effects of dietary supplementation with RPT on neuroendocrine and acute-phase markers in yaks (ng/L).

Item	Treatment ^1^			SEM	*p*-Value
	CON	RPT20	RPT40		
SAA	14.5	16.3	13.2	0.791	0.296
CRP	7.18	6.84	7.03	0.100	0.396
EPI	28.7	32.7	27.6	1.66	0.446
ACTH	20.4	26.4	20.7	3.32	0.733
COR	44.0	44.2	46.0	2.71	0.952

RPT = rumen-protected taurine; SAA = serum amyloid A; CRP = C-reactive protein; EPI = epinephrine; ACTH = adrenocorticotropic hormone; COR = cortisol. *n* = 6. ^1^ CON means a basic diet without RPT; RPT20 means a basal diet with daily supplementation of 20 g of RPT; RPT40 means a basal diet with daily supplementation of 40 g of RPT.

**Table 6:** Effects of dietary supplementation with RPT on immune function in yaks (ng/L).

Item	Treatment ^1^			SEM	*p*-Value
	CON	RPT20	RPT40		
IL-1	38.4	45.5	38.7	3.35	0.651
IL-6	44.8	41.3	40.5	3.22	0.863
TNF-α	39.9	38.2	27.2	3.85	0.470
IFN-γ	72.2	81.2	69.5	3.74	0.444
IgA	35.7 ^b^	53.9 ^a^	38.2 ^b^	3.18	0.0293
IgG	28.0 ^b^	43.7 ^a^	33.4 ^ab^	2.59	0.0428
IgM	23.8	28.0	21.9	2.02	0.476

RPT = rumen-protected taurine; IL-1 = interleukin-1; IL-6 = interleukin-6; TNF-α = tumor necrosis factor; IFN-γ = interferon-gamma; IgA = immunoglobulin A; IgG = immunoglobulin G; IgM = immunoglobulin M. ^a,b^ Within a column, means with different superscripts differ, *p* < 0.05. *n* = 6. ^1^ CON means a basic diet without RPT; RPT20 means a basal diet with daily supplementation of 20 g of RPT; RPT40 means a basal diet with daily supplementation of 40 g of RPT.

## Data Availability

The original contributions presented in this study are included in the article. Further inquiries can be directed to the corresponding author.
